# Structure Determination of F_4_TCNQ on Ag(111): A Systematic Trend in Metal Adatom Incorporation

**DOI:** 10.1021/acsomega.4c04860

**Published:** 2024-07-10

**Authors:** Archie L. Hobson, Hadeel Hussain, Philip J. Mousley, David A. Duncan, Mona Braim, Giovanni Costantini, Christopher Nicklin, D. Phil Woodruff

**Affiliations:** † Department of Physics, University of Warwick, Coventry CV4 7AL, U.K.; ‡ Diamond Light Source, Harwell Science and Innovation Campus, Didcot OX11 0DE,U.K.; § School of Chemistry, University of Birmingham, Edgbaston, Birmingham B15 2TT, U.K.

## Abstract

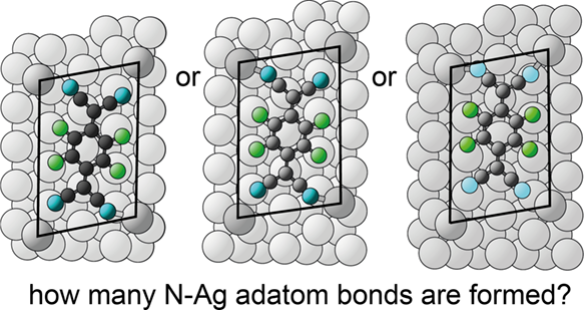

A structure determination of the commensurate phase formed by 7,7,8,8-tetracyano-2,3,5,6-tetrafluoroquinodimethane (F_4_TCNQ) absorbed on Ag(111) is reported. Initial characterization was performed using low-energy electron diffraction and synchrotron radiation photoelectron spectroscopy, with quantitative structural data being provided by normal incident X-ray standing waves (NIXSW) and surface X-ray diffraction (SXRD). NIXSW data show the F_4_TCNQ molecule to adopt a “twisted” conformation on the surface, previously found to be associated with metal adatom incorporation into a 2d-metal–organic framework for F_4_TCNQ on Au(111), Ag(100), and Cu(111). SXRD results provide direct evidence of the presence of Ag adatoms that are found to occupy near-bridge or fcc hollow sites with respect to the underlying surface, at an adsorption height of 2.69 ± 0.10 Å. The results show a consistent pattern of behavior for F_4_TCNQ adsorption on the (111) surfaces of Cu, Ag, and Au.

## Introduction

**Figure 1:**
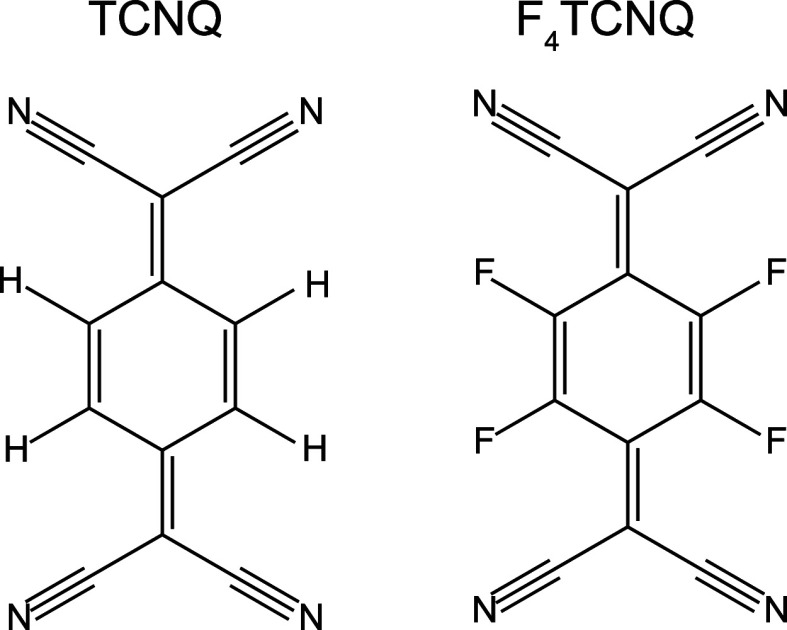
Structural formulas of TCNQ and F_4_TCNQ.

The electronic properties of devices based on organic semiconductors can be strongly influenced by the properties of their interfaces at conductive electrodes; this has led to a significant number of surface science studies of related model systems. 7,7,8,8-tetracyanoquinodimethane (TCNQ) and its fluorinated variant, F_4_TCNQ, (Figure 1) are electron acceptor molecules of particular interest as molecular dopants or for work function engineering in organic devices.^1−3^ As such, there have been several studies of their adsorption on mainly coinage metal surfaces, particularly with a (111) orientation, as described more fully below.

Spectroscopic studies clearly demonstrate charge transfer from the metal surface,^4−10^ leading to rehybridization of the intramolecular bonding that relaxes the rigidity of the planar gas-phase molecule.^11^ Until recently, however, the only quantitative information on the detailed structure of these adsorbed molecules had been provided by density functional theory (DFT) calculations, which have predicted that the adsorbed molecule adopts an inverted bowl or umbrella conformation, with the cyano N atoms bonding to the surface while the central quinoid ring is parallel to, but up to 1.4 Å further from, the surface (e.g., refs (8,12−15)). All of these calculations have assumed that the adsorbate does not induce a reconstruction of the metal surface. Most recently, experimental quantitative structural techniques have been applied to several of these adsorption systems, leading to results that challenge this previously established wisdom. Initially, normal incidence X-ray standing wave (NIXSW)^16^ experiments were used to determine the heights above the surface of the chemically inequivalent atoms within the molecule. In the case of an ordered commensurate phase of TCNQ on Ag(100) the results^17^ are consistent with the adsorption of a symmetric molecule, bent into an inverted bowl conformation, as predicted by earlier DFT calculations. By contrast, NIXSW data for TCNQ adsorption on Ag(111),^18^ and for F_4_TCNQ adsorption on Ag(100),^17^ on Au(111)^19^ and on Cu(111),^20^ are not consistent with this picture. In particular, they show that the N atoms of the molecule must occupy (at least) two distinctly different heights above the surface, leading to a twisted molecular conformation. Dispersion-corrected DFT calculations have shown that this behavior can be reconciled with the uppermost pair of cyano-groups being coordinated to metal adatoms from the substrate through the nitrogen atom, with the remaining pair coordinated to the underlying substrate metal atoms. The incorporation of metal adatoms into the molecular overlayer results in the formation of a two-dimensional metal–organic framework (2D MOF).

Unfortunately, NIXSW is not able to provide *direct* evidence of the presence of metal adatoms; the chemical shift in the C 1s XPS allows the chemically inequivalent C atoms in F_4_TCNQ to be clearly distinguished, but any chemical shift between core level photoemission from the metal adatoms and metal substrate is too small to allow the determination of the adatom sites. In the case of the Au(111)-F_4_TCNQ system scanning tunneling microscopy (STM) images show protrusions that have been interpreted as due to Au adatoms,^21^ while there is some evidence of similar protrusions in STM images of F_4_TCNQ on Cu(111)^20^ and Ag(111),^3^ although no such features are found in STM images of the Ag(111)-TCNQ system.^18^ It is widely acknowledged in the literature that the existence (or absence) of these characteristics in STM images should not be considered conclusive evidence of the inclusion (or exclusion) of adatoms in the molecular overlayers (e.g., refs (18,22,23)). By contrast, direct evidence of the presence and location of the metal adatoms can be obtained from surface X-ray diffraction (SXRD). The small X-ray scattering cross-section for low atomic number atoms that comprise the adsorbed molecules means that SXRD is not well-suited to determine pure molecular overlayer structures, but the technique is sensitive to the location of atoms of higher atomic number, such as metal adatoms; this is the basis of the so-called “heavy atom” technique used in macromolecular crystallography. Using this approach, SXRD has been employed to determine the presence and location of metal adatoms in the Au(111)-F_4_TCNQ^19^ and Ag(111)-TCNQ^24^ adsorption systems.

The fact that F_4_TCNQ adsorption on both Au(111) and Cu(111) leads to metal adatom incorporation into 2D MOF overlayers strongly suggests that the same behavior may be expected for F_4_TCNQ adsorption on Ag(111). Here we present the results of a structural investigation of this system, using both NIXSW and SXRD, that demonstrates that this is, indeed, the case, identifying the conformation and location of the adsorbed molecules and the presence and location of the Ag adatoms within the precision of the SXRD technique.

## Methods

Initial characterization of the adsorption of F_4_TCNQ on Ag(111) was performed by low-energy electron diffraction (LEED) in the UHV endstation EH2 on Beamline I07 at the Diamond Light Source,^25^ and by X-ray photoelectron spectroscopy using both soft and hard X-rays at the UHV endstation of Beamline I09^26^ before and after molecular deposition. The Ag(111) crystal was cleaned in situ using standard cycles of argon ion sputtering followed by annealing to 450 °C. F_4_TCNQ molecules were deposited onto the Ag(111) single crystal surface from a simple thermal molecular evaporation source operated at a temperature of ∼100 °C with the sample at nominal room temperature after cooling for 1–2 h following annealing. NIXSW measurements to monitor the X-ray absorption at the C, N, and F atoms of F_4_TCNQ, were performed by measuring the intensity of the C 1s, N 1s, and F 1s photoelectron spectra, as the photon energy was stepped through the (111) Bragg condition (*h*ν ∼ 2972 eV) at near-normal incidence to the Ag(111) sample. In the case of the C 1s spectra, individual spectra were fitted with multiple chemically shifted components to distinguish the signals from the chemically inequivalent C atoms. Fitting of the NIXSW absorption profiles to extract the structural parameters included taking account of the nondipolar effects on the angular dependence of the photoemission, using values for the backward-forward asymmetry parameter *Q*,^27^ obtained from theoretical angular distribution parameters.^28^ SXRD measurements of the intensities of the diffracted beams, *hk* were recorded as a function of momentum transfer perpendicular to the surface, *l*, at a photon energy of 19 keV and a grazing incidence angle of 1°. Modeling the SXRD data to determine the best-fit structural model was performed using the ANA-ROD program.^29^

## Results

### Surface Characterization

**Figure 2:**
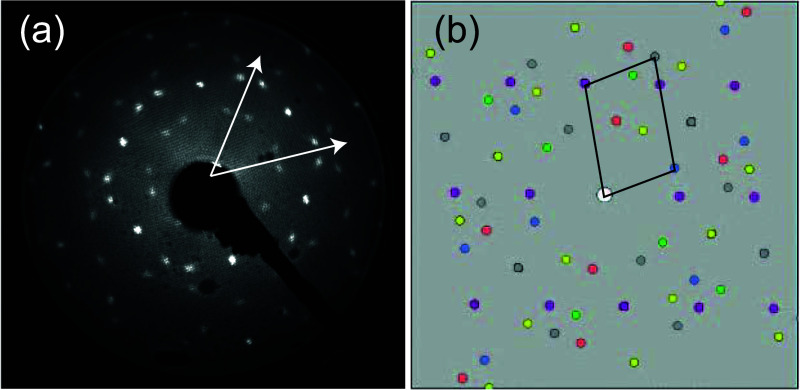
(a) LEED pattern of the  phase formed by F_4_TCNQ on Ag(111) recorded at an electron energy of 30 eV. The arrows indicate <110> azimuthal directions (b) LEEDpat simulation of the expected LEED pattern from this phase. The different colors correspond to different rotational and mirrored domains; the reciprocal unit mesh of one domain is superimposed.

Deposition of F_4_TCNQ onto the Ag(111) surface led to the formation of a commensurate ordered overlayer as indicated by the LEED pattern shown in Figure 2a. Comparison with a simulated pattern (Figure 2b) using the LEEDpat program^30^ identifies the overlayer matrix as . This mesh is consistent with a previously published STM image of F_4_TCNQ adsorbed on Ag(111).^3^ Notice that the symmetry of this mesh is lower than that of the underlying Ag(111) surface, which leads to 6 different rotational and mirrored domains of the overlayer. The predicted LEED patterns of the different domains are shown in different colors in Figure 2b, but note that half of the diffracted beams from each domain overlap with beams from at least one other domain on the surface; this is taken into account by the ROD computer program used in the SXRD data analysis.

**Figure 3:**
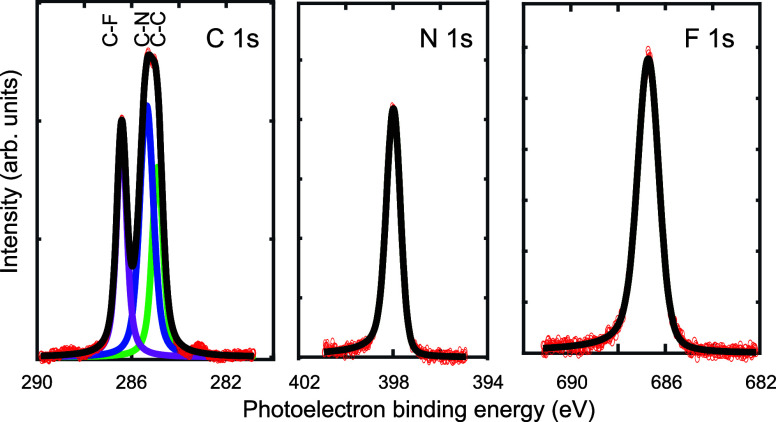
C 1s, N 1s, and F 1s SXP spectra recorded for the ordered overlayer of F_4_TCNQ on Ag(111). Individual data points are shown in red, while the overall fit is shown as a black line. The C 1s spectrum shows the three chemically shifted components associated with C–F (red), C–N (blue), and C–C (green) local environments. The photon energies used were 435 eV (C 1s), 550 eV (N 1s), and 850 eV (F 1s). Binding energies are relative to the Fermi edge measured at the same photon energies.

Soft X-ray C 1s, N 1s, and F 1s photoelectron spectra (SXPS) are shown in Figure 3. The N 1s spectrum shows a single peak, indicating that all N atoms occupy very similar chemical environments. The F 1s SXP spectrum also comprises a single peak, whereas the C 1s spectrum can clearly be resolved into three components corresponding to the C–F, C–C, and C–N atomic environments, respectively.

### Structural Measurements

To determine the adsorption height of the molecular component atoms and thus also the molecular conformation of F_4_TCNQ on Ag(111), (111) NIXSW measurements were carried out on the  phase. The variation of the photoemission intensity from each of the C 1s, N 1s, and F 1s components as a function of photon energy, when scanned through the Ag (111) Bragg condition, can be uniquely fitted by two parameters, the coherent position, *p*, and coherent fraction, *f.*(16) The coherent fraction can be regarded as an order parameter; in the ideal case in which all atoms of a particular chemical character occupy identical sites, with no static or dynamic disorder, the coherent fraction is unity. In this case, the coherent position is the height of these absorbing atoms above the underlying Bragg planes, in units of the bulk interlayer spacing, *d*. In practice, the value of the coherent fraction is reduced by disorder, but if the overlayer comprises only a single molecular layer, this disorder cannot reduce the coherent fraction below about 0.7.^31^ Lower values of the coherent fraction indicate that atoms, although of the same chemical environment must occupy at least two heights relative to the scatterer planes. Table 1 shows the values of the coherent fraction and position obtained from fitting the experimental NIXSW data from the different chemically distinct atomic species, while the absorption profiles and the fits are shown in Figure S1. Also shown in the table are values of the parameter *D* = (*p* + *n*)*d*, the height of the atoms above the Bragg planes in Ångström units; *n* is an integer (usually 0 or 1) chosen to ensure that the value of *D* is consistent with physically reasonable interatomic distances. Measurements taken from three separate preparations of the surface led to consistent results.

**Table 1:** NIXSW Fitting Parameter Values for the Experimental Data for F_4_TCNQ on Ag(111)

	N	C–F	C–N	C–C	F
*f*	0.31 ± 0.03	0.70 ± 0.06	0.53 ± 0.07	0.61 ± 0.05	0.44 ± 0.03
*p*	0.15 ± 0.02	0.31 ± 0.03	0.20 ± 0.04	0.37 ± 0.03	0.35 ± 0.02
*D* (Å)	2.70 ± 0.05	3.08 ± 0.07	2.82 ± 0.10	3.22 ± 0.07	3.17 ± 0.05

The relatively high *f* value for the C atoms (C–F) in the quinone ring indicates that this ring is parallel to the surface, but the very low *f* value for the N atoms clearly indicates that the N atoms must occupy at least two different heights above the surface. This necessarily means that the C–N, and to a lesser extent the C–C atoms must occupy slightly different adsorption heights, consistent with the slightly reduced *f* values for these atoms. These results are closely similar to those obtained previously for F_4_TCNQ on Ag(100),^32^ Cu(111)^20^ and Au(111)^19^ and for TCNQ on Ag(111),^18^ indicating that the molecule adopts a twisted conformation on the surface, although the fact that the C–C atoms are marginally higher than the C–F atoms (by 0.14 ± 0.10 Å) differs from these earlier studies. The origin of the low coherent fraction for the fluorine is unclear, but this has also been seen in the earlier studies of F_4_TCNQ adsorption.^19,20,32^ In some cases a chemically shifted F 1s component was attributed to the presence of atomic fluorine (at a binding energy of 682 eV^33^) due to radiation damage (e.g., ref (20)), but no such shifted peak was seen in the present study. We can only surmise that radiation damage leads to a separate molecular F species that does not have a detectable 1s XPS chemical shift relative to F_4_TCNQ and does not have C atoms at significantly different heights to those of the coadsorbed intact F_4_TCNQ.

In the previous studies in which NIXSW data indicated that adsorbed TCNQ or F_4_TCNQ adopted a twisted conformation,^18,20^ DFT calculations found that this result, and the detailed measured NIXSW adsorption profiles, could be reconciled with the effect of coadsorbed metal adatoms. Two different N atom heights arise because some N atoms bond to these adatoms, while other N atoms are bonded to the underlying substrate atoms. In the present system, we do not have the benefit of the results of prior DFT calculations, but SXRD offers a method to determine the presence and location of any Ag adatoms experimentally.

In a standard X-ray diffraction structure determination of a 3D bulk crystal, the experimental data set comprises the intensity of many individual *hkl* diffracted beams. In SXRD, in which the sample is only 2D-periodic, momentum is conserved parallel to the surface but not perpendicular to the surface. The momentum transfer perpendicular to the surface, represented by *l*, is a continuous variable rather than a set of discrete values. The reciprocal lattice of the 3D crystal is replaced by a reciprocal mesh, with infinite “rods” perpendicular to this mesh, passing through the reciprocal mesh *hk* points. A SXRD data set thus comprises diffracted intensities measured as a function of the continuous parameter *l* at different *hk* values. Most molecular overlayers have a larger surface mesh than the underlying substrate, as is the case in this study. This larger real-space mesh means that the reciprocal mesh of the overlayer must be smaller than that of the substrate, leading to “extra” *hk* values. By convention, the labeling of *h* and *k* is relative to the unit reciprocal mesh of the substrate. The intensities of integral *hk* beams as a function of *l* therefore contain intense peaks due to bulk diffraction; these intensity scans are known as *crystal truncation rods* (CTRs), and their intensities are determined by the structure of both the surface and the underlying bulk. By contrast, the “extra” *hk* beams have fractional indices (rational fractions if the overlayer is commensurate) and these *fractional order rod* (FOR) scans have intensities determined *only* by the structure of the surface layer(s) that have the larger real-space unit mesh.

In the present study, the collected data set comprised the *l*-dependence of 14 CTRs (including the (00) reflectivity), together with the *l*-dependence of 8 FORs, and additionally 23 “in-plane” fractional order beam intensities at a low value of *l* (*l* = 0.3). The FOR intensities were all taken from a single domain. As each of the 6 rotational/mirror domains is equivalent, only one of them needs to be measured. The structure determination was then achieved by iteratively comparing the simulated intensities from a “working” model with the experimental data using χ^2^ minimization to identify the best-fit structure.

In the two previous similar SXRD studies of Au(111)-F_4_TCNQ^19^ and Ag(111)-TCNQ,^22^ the results of DFT calculations provided the starting structure for the modeling. In the present case, no DFT results are available, thereby providing a more challenging test of the methodology. The primary objective of the structural search was to distinguish between the adatom and no-adatom models, but also to determine the height and lateral registry of the overlayer relative to the substrate, as well as establish the amplitude of any rumpling of the outermost Ag(111) layers. SXRD is not sufficiently sensitive to the exact relative locations of individual weakly scattering C, N, and F atoms to determine the molecular conformation, so all calculations assumed a rigid adsorbed F_4_TCNQ molecule having the twisted conformation found in the combined NIXSW, SXRD, and DFT investigation of the Au(111)-F_4_TCNQ system.^19^ The results of the SXRD simulations did, however, prove to be sensitive to the surface rumpling and the location and azimuthal orientation of the adsorbed molecules, as well as both the presence and location of an Ag adatom.

Two primary models were tested against the SXRD calculations: one with a single Ag adatom per surface unit mesh and one with no Ag adatoms per surface unit mesh. Both models contained a single F_4_TCNQ molecule per surface unit mesh. A third model, in which each surface unit mesh contained two Ag adatoms in addition to the one F_4_TCNQ molecule was explored, but gave a much worse fit to the experimental data as judged by the χ^2^ value, as described below. For both the adatom and no-adatom models, the initial structure was built using the unit mesh dimensions provided by the LEED measurements, each unit mesh being assumed to contain a single F_4_TCNQ molecule as indicated by the STM images.^3^ Both models were placed on top of 3 surface layers of Ag atoms with displacement parameters perpendicular to the surface applied to individual Ag atoms, and each layer as a whole. This allowed the possible effects of rumpling and relaxation of the outermost Ag layers to be explored. These 3 layers were modeled on top of bulk Ag(111), the atoms of which were not allowed to vary in position.

Notice that the intensities of fractional order beams are determined entirely by the structure of the surface; i.e., those atoms having the periodicity of the overlayer. As such, the registry of the surface and substrate can normally only be determined by analysis of the CTRs, which are influenced by scattering from both the surface and the bulk. However, the measured CTRs (Figure S2) are essentially “bulk-like”, with no significant structural changes to the anti-Bragg regions of the scans when compared with scans from clean Ag(111). This is a consequence of the very low coverage of the molecules (and the Ag adatoms) together with the low scattering cross sections of the low atomic number elements that constitute the molecules. As such, the CTRs alone did not provide a sound basis to differentiate between different structural models, although they did show a weak dependence on the Ag adatom location. The (00) CTR (reflectivity) does show some weak structure at ∼ *l* = 1.2, which was found to be primarily sensitive to the rumpling and relaxation of the outermost Ag layers but was also influenced by out-of-plane displacements of the F_4_TCNQ molecule. However, by including the three outermost Ag layers within the model of the surface, analysis of the fractional order diffraction data alone does provide information on the registry of the molecular overlayer (and adatom) relative to these three layers. In part, this sensitivity relies upon two assumptions: (1) that at least some of the surface Ag atoms are rumpled to a measurable degree and (2) that the lateral sites of the outermost three layers of Ag atoms are in the bulk continuation sites and have not undergone any significant lateral reconstruction. The intensities of each FOR scan relative to one another were found to be most sensitive to adatom height and lateral position, with minimal impact from the location of the F_4_TCNQ molecules. Rumpling of the top two layers of substrate Ag atoms was found to be important in determining the shape of the FOR *l*-scans but had less influence on the relative intensities of the different rod scans. By contrast, the in-plane FOR intensities showed significant sensitivity to the orientation and location of the F_4_TCNQ molecule.

**Figure 4:**
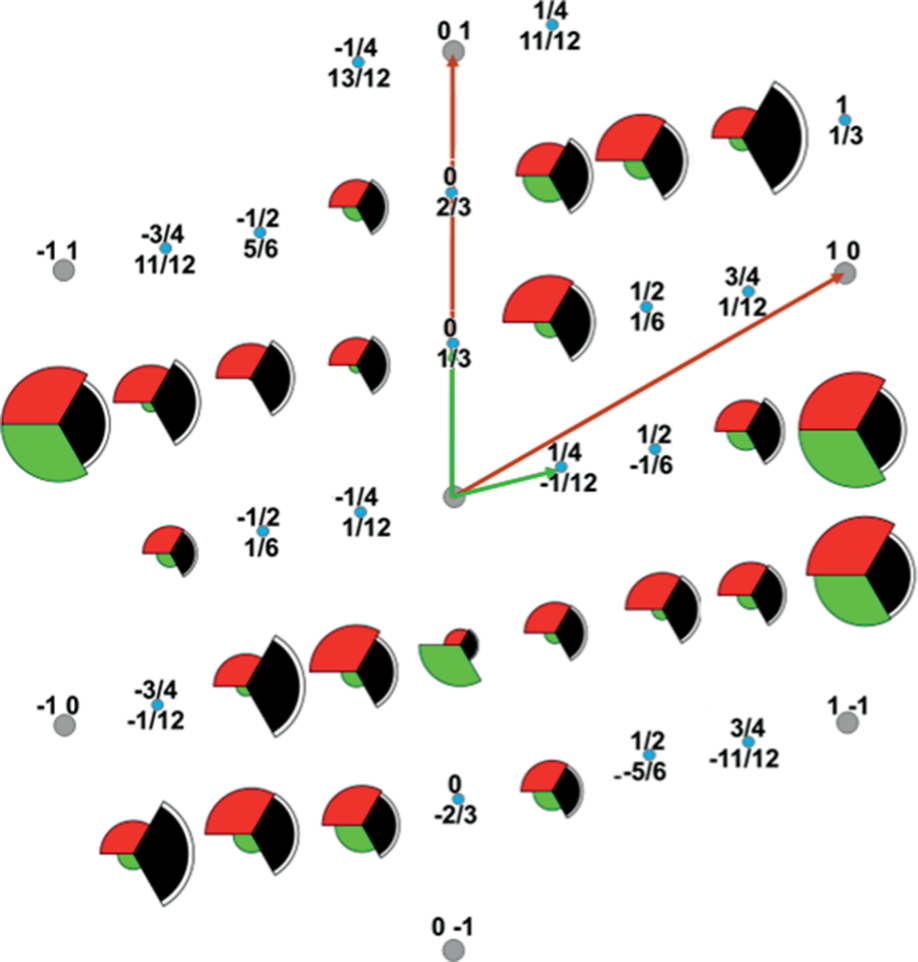
Comparison of the measured experimental in-plane fractional order beam structure factors (black) with the results of simulations for the best-fit adatom model (red) and the best-fit no-adatom model (green). The areas of the colored arcs correspond to the measured and predicted structure factors. The white rims of the black experimental sectors indicate the estimated error bars. Fractional order diffracted beams for which no intensity measurements are available are represented by small blue filled circles, while integral-order beam locations are represented by small gray filled circles. The primitive translation vectors of reciprocal nets of the substrate and overlayer are shown by the gray and blue arrows, respectively.

A comparison of the experimental FOR rod scan data and simulations based on the best-fit adatom and no-adatom models is shown in Figure S3. This subjective comparison shows that the adatom model provides a slightly better fit to the experimental data for both the absolute values of the predicted structure factors and the shape of their *l*-dependence, although the differences are rather small. However, the preference for the adatom model is far stronger in a comparison of the experimental “in-plane” fractional order structure factors with simulated values for the two models, shown in Figure 4. For the majority of the diffracted beams, the predicted structure factors (the square root of the intensities) for the no-adatom model is significantly smaller than for the adatom model, the adatom model clearly giving a better fit to the experimental data. A comparison of χ^2^ values for the in-plane data alone shows a clear preference for the adatom model as detailed below.

Four basic single adatom models were considered initially with the Ag adatom occupying atop, bridge, fcc hollow, and hcp hollow sites. For each of these models, a range of different azimuthal orientations of the F_4_TCNQ molecule were considered, the lateral position and height of the molecule for each orientation being adjusted to give the best agreement with the experimental data; the criterion for this best agreement was the lowest value of χ^2^ for the complete data set, which we refer to below as the global χ^2^. This criterion clearly favors the adatom models over the no-adatom model, with the adatom in either the fcc hollow site (χ^2^ = 1.23) or the bridge site (χ^2^ = 1.22). Assuming that a 5% variation in χ^2^ relative to the lowest value defines the range of acceptable structures, the models with Ag adatoms in atop and hcp hollow sites (χ^2^ values of 1.72 and 1.73, respectively) can be excluded, as can the no-adatom model (χ^2^ = 1.48). Searches of adatom sites displaced from the four high-symmetry sites revealed a lowest global χ^2^ value of 1.18 for a site approximately 0.25 Å from the exact bridge site; although this is the lowest value of the global χ^2^, it differs by less than 5% from the values for the exact bridge and fcc hollow site, so there is some ambiguity in the exact adatom site. The values of two alternative χ^2^ values were also considered, namely those based only on comparison of the diffracted intensities of the fractional order beams, which might be expected to be more sensitive to the details of the surface structure. These were FOR χ^2^, comparing only the intensities recorded in the fractional order rod scans, and in-plane χ^2^, comparing only the in-plane intensity measurements.

**Figure 5:**
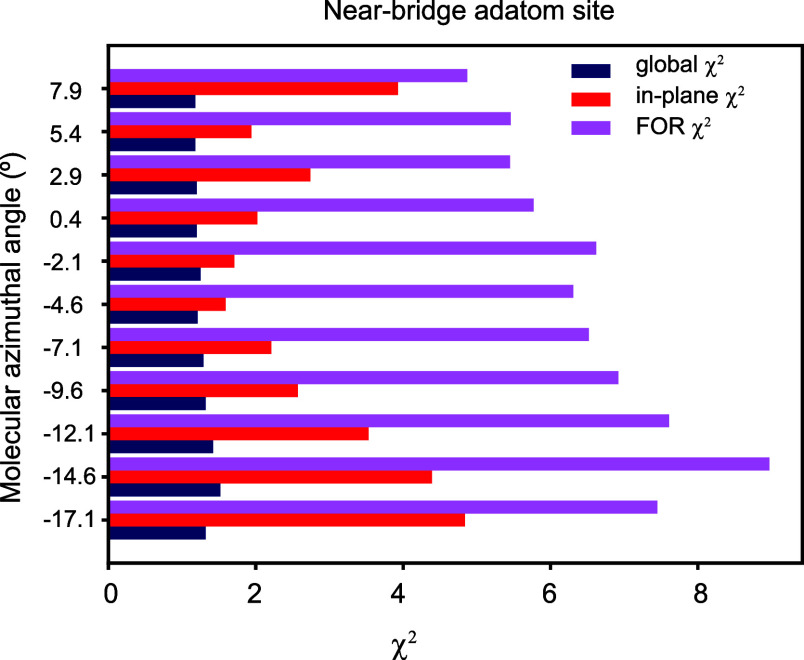
Variation of the three different χ^2^ values as a function of the azimuthal angle of the F_4_TCNQ molecule for the structural model in which the Ag adatoms occupy the most favored near-bridge sites. The azimuthal angle is defined as the angle between the central axis of the molecule and the longer primitive translation vector that defines the surface unit mesh (see Figure 6a).

Figure 5 shows the variation of these three different χ^2^ quantities as a function of the azimuthal orientation of the molecule within the unit mesh, for the model in which the Ag adatom occupies the most favored near-bridge site. Similar trends were found for the alternative high-symmetry adatom sites. The global χ^2^ value is only weakly dependent on the location and orientation of the F_4_TCNQ molecule. This is a consequence of the fact that much the largest number of measured intensity data points are in the CTRs, and the CTRs are least sensitive to the structure of the surface layers. By contrast, there are particularly strong variations in the values of the in-plane χ^2^ as the molecular orientation is varied. Assuming that a 5% variation in χ^2^ relative to the lowest value defines the range of acceptable structures, the precision of the azimuthal angle using in-plane χ^2^ is approximately ±2°. By contrast, using the global χ^2^, the precision is at best ±10°. This strong sensitivity of the in-plane intensities to the relative positions of the atoms within the surface structure is consistent with the fact that a sufficiently large set of in-plane intensities is the basis of the construction of a Patterson map, which reveals the interatomic vectors of the surface structure projected onto the surface plane.

The preference for the adatom model indicated particularly by the comparison of the in-plane data shown in Figure 4 is reinforced by the quantitative comparison afforded by the values of the normalized in-plane χ^2^. Specifically, the values for the alternative adatom models fell in the range of 1.58 to 1.94 whereas the value for the no-adatom model was 15.12. Table 2 shows the adsorption heights of the adsorbed molecule and the Ag adatom of the best-fit SXRD structures, together with the rumpling amplitudes of the outermost two Ag(111) layers. In fact, the precision of these measurements is such that the rumpling of the second layer is barely significant. No rumpling of the third layer was found in the best-fit model. Despite the weak scattering of the low atomic number of the atoms in the F_4_TCNQ molecule, the height of the molecule found in the SXRD analysis is consistent with the value given by the NIXSW data (formally they differ by 0.14 ± 0.12 Å).

**Table 2:** Values of the Structural Parameters Obtained from the NIXSW Data (Table 1) and from the SXRD Data Analysis for the Two Alternative Structural Models^a^

	NIXSW	SXRD – adatom	SXRD– no-adatom
F_4_TCNQ height	3.08 ± 0.07 Å	3.22 ± 0.09 Å	3.13 ± 0.10 Å
adatom height		2.69 ± 0.10 Å	
rumpling amplitude 1st layer		0.53 ± 0.20 Å	0.56 ± 0.20 Å
rumpling amplitude 2nd layer		0.14 ± 0.20 Å	0.16 ± 0.20 Å

a The height of the F_4_TCNQ given here corresponds to the height of the quinone ring of CF carbon atoms. NIXSW distinguishes these C atoms from other atoms in the molecule, but the SXRD calculations are of a molecule of a fixed conformation, varying the positions relative to the underlying substrate, so while the quoted precision of the NIXSW value is of the height of this particular part of the molecule, whereas the SXRD precision value is of the height of the whole (rigid) molecule. The SXRD precision estimates for the molecule and adatom heights are determined by the range of individual parameter values that lead to a value of χ^2^ within 5% of the best-fit value.^19^ In each case, the height is given relative to a bulk-terminated outermost Ag(111) layer, thereby taking account of the small (0.03 Å) change in outermost layer spacing found in the SXRD analysis. Rumpling amplitudes correspond to the height differences of the lowest and highest Ag atoms within each layer.

The large rumpling amplitude of the outermost Ag(111) layer of the (preferred) adatom model found here is similar to those found in our SXRD studies of TCNQ adsorption on Ag(111) and F_4_TCNQ on Au(111). These values are significantly larger than the values found in DFT calculations for the Ag(111)-TCNQ and the Au(111)-F_4_TCNQ system.^19^ The origin of this discrepancy is unclear. DFT calculation uses a thin slab (typically no more than 3 or 4 atomic layers) to represent the surface and the underlying bulk, so some discrepancy might be expected, although one might surmise that calculations on a thin slab would lead to larger, rather than smaller, rumpling amplitudes. Of course, it is also important to note that there is an absence of alternative experimental determinations of these parameters for the adsorption of relatively large molecules on surfaces.

**Figure 6:**
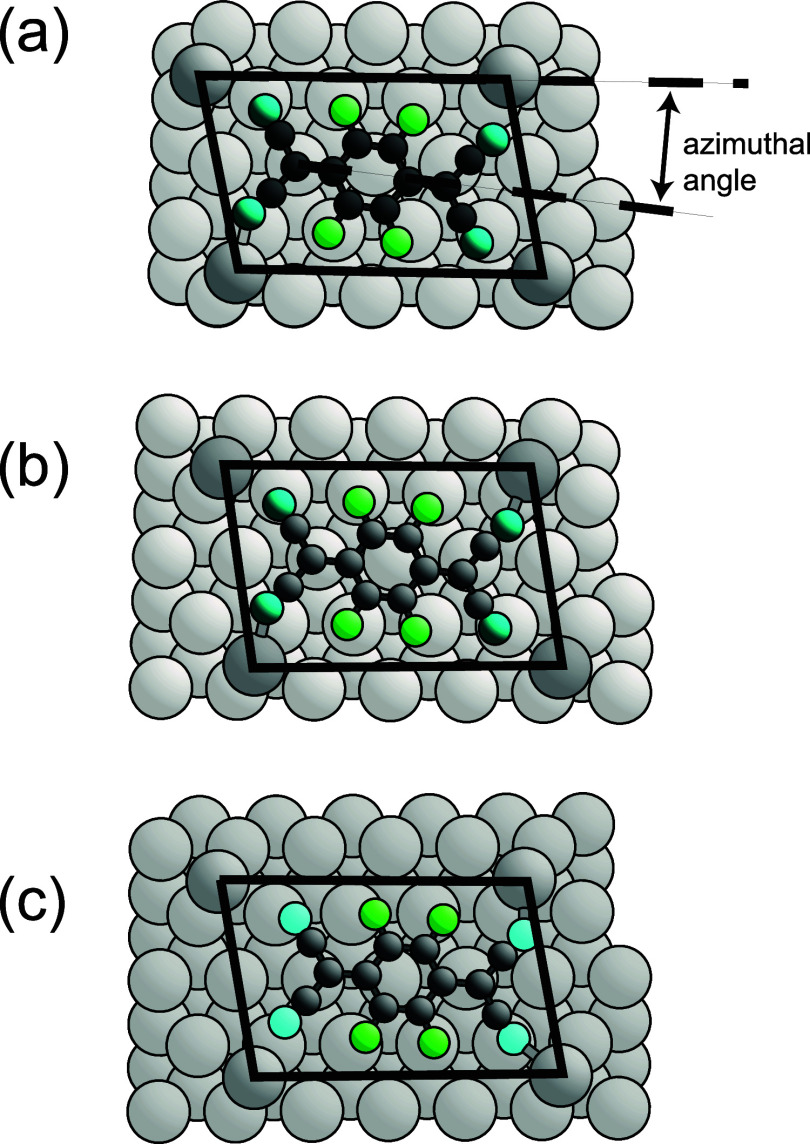
Plan view of Ag(111) F_4_TCNQ structures as determined by SXRD. Atom coloring: Ag substrate, light gray; Ag adatom, dark gray; C, black; F, green; and N, blue. (a) shows the near-bridge structure at the azimuthal angle corresponding to the lowest value of the in-plane χ^2^_._ (b) shows the near-bridge structural models at a molecular azimuthal angle that differs from that in (a) by 2.5°. (c) shows the best-fit exact bridge structural model in which the azimuthal angle of the molecules is that same as in (b). N–Ag adatom bonds are shown for interatomic distances less than 2.5 Å. The black lines show the surface unit mesh.

In our previous investigations of F_4_TCNQ adsorption on Au(111) and TCNQ adsorption on Ag(111), DFT calculations have clearly identified the in-plane molecule-adatom bonding, thereby indicating that the surface layer is a 2D MOF. A SXRD structural study cannot identify the bonding character, which can only be inferred from the N-adatom interatomic distances. However, the precision of our determination of these distances is only approximately ±0.25 Å, much too large to characterize chemical bonds. This problem is illustrated by Figure 6, which shows plan views of three different structures that all fall within the range of acceptable structures as defined by 5% variations in the values of the global χ^2^. Bonds are shown for N–Ag atom distances of less than 2.5 Å. Which cyano N atoms appear to bond to which Ag adatoms differ in the three models and highlight the difficulty of identifying the specific bonding that would be associated with a 2D MOF.

As remarked above, the two-adatom model can be clearly rejected on the basis of its global χ^2^ value of 2.70 (the value of the in-plane χ^2^ of 12.56 is even more conclusive). A plan view of this best-fit two-adatom structure is shown in Figure S4, which illustrates a further problem with this model. The presence of the second Ag adatom in the surface unit mesh constrains the space available for the F_4_TCNQ molecule such that all four cyano N atoms fall within bonding distances to Ag adatoms; in this case, one would expect all the N atoms to have essentially the same height above the surface, inconsistent with the NIXSW results.

At first glance, the preference for the single Ag adatom to occupy the low symmetry off-bridge site seems surprising, although we have noted that occupation of the exact bridge site or the fcc hollow also falls within the range of acceptable values of χ^2^. Ag adatoms on a clean Ag(111) surface would certainly be expected to occupy fcc hollow sites. However, the preferred location of the Ag adatoms is determined by the registry of the complete F_4_TCNQ-Ag adatom MOF, with the lower cyano N atoms bonding to the underlying Ag(111) surface. As shown in Figure 6, the off-bridge adatom registry leads to the lower cyano N atoms being close to atop sites relative to the underlying Ag(111) surface, which may lead to this registry being favored.

## Results and Discussion

A structural investigation of the Ag(111)  F_4_TCNQ structure has been achieved using a combination of the NIXSW and SXRD measurements at the Diamond Light Source. NIXSW data clearly show that the molecules do not adopt the symmetric inverted bowl conformation favored by DFT calculations performed assuming an unreconstructed Ag(111) surface.^14,15^ Instead, they adopt a twisted conformation with the bonding N atoms occupying (at least) two distinctly different heights above the surface. Previous studies of TCNQ adsorbed on Ag(111),^22^ and F_4_TCNQ on Au(111)^19^ have shown that this molecular conformation is due to the presence of metal adatoms in the molecular overlayer to form a 2D MOF. Our SXRD investigation of the present Ag(111)-F_4_TCNQ system clearly shows that similar Ag adatom incorporation occurs on this surface. Unlike these previous cases, no results of prior DFT calculations are available for this surface reconstruction; the structure reported here has been determined entirely from quantitative experimental structural data. Specifically, the SXRD analysis demonstrates the presence and preferred location of the Ag adatoms and F_4_TCNQ molecule in the overlayer and provides quantitative information on the heights of the adsorbate components and on the induced rumpling of the outermost Ag layers. However, the precision in the determination of the interatomic distances in the surface is insufficient to distinguish alternative models of the adatom-molecule bonding within the surface layer. Nevertheless, these results demonstrate a consistent pattern of behavior for F_4_TCNQ adsorption on Au(111), Ag(111), and Cu(111);^20^ in each case, the molecular adsorption is accompanied by the incorporation of metal adatoms.

## Supporting Information Available

Additional experimental results: NIXSW data; comparison of experimental and theoretical CTRs and FORs (PDF)
