# Supplementary material for: Structure Determination of F4TCNQ on Ag(111): A Systematic Trend in Metal Adatom Incorporation
Source: ACS Omega. 2024 Jul 10;9(29):32193–200. doi: 10.1021/acsomega.4c04860 (PMC11270728; doi:10.1021/acsomega.4c04860)
Supplement: Supplementary file 1 — ao4c04860_si_001.pdf [file ao4c04860_si_001.pdf]

SUPPLEMENTARY INFORMATION

Structure Determination of F_4TCNQ on Ag(111): a Systematic Trend in Metal Adatom Incorporation

Archie L. Hobson[1,2], Hadeel Hussain[2], Philip J. Mousley[2], David A. Duncan[2], Mona Braim[1], Giovanni Costantini[,3], Christopher Nicklin[2], D. Phil Woodruff[1*]

[1]*Department of Physics, University of Warwick, Coventry CV4 7AL, UK*
[2]*Diamond Light Source, Harwell Science and Innovation Campus, Didcot, OX11 0DE,*
[3]*School of Chemistry, University of Birmingham, Edgbaston, Birmingham B15 2TT, UK*

[*] email d.p.woodruff@warwick.ac.uk

NIXSW

Figure S1 shows the experimental NIXSW absorption profiles together with the best-fit results corresponding to the coherent fraction and position values reported in Table 1 of the main manuscript.

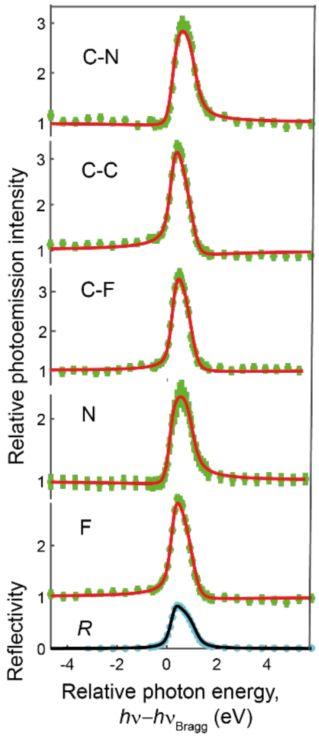

Figure S1 individual C 1s, N 1s and F 1s experimental NIXSW profiles (green data points) and best-fit theory curves (red lines) corresponding to the coherent fraction and coherent position values reported in Table 1. Also shown is the reflectivity, *R*.

Figures S2 and S3 shows comparisons of the experimental CTRs and FORs, respectively, with the results of simulations for the best-fit adatom and best-fit no adatom structural models.

SXRD

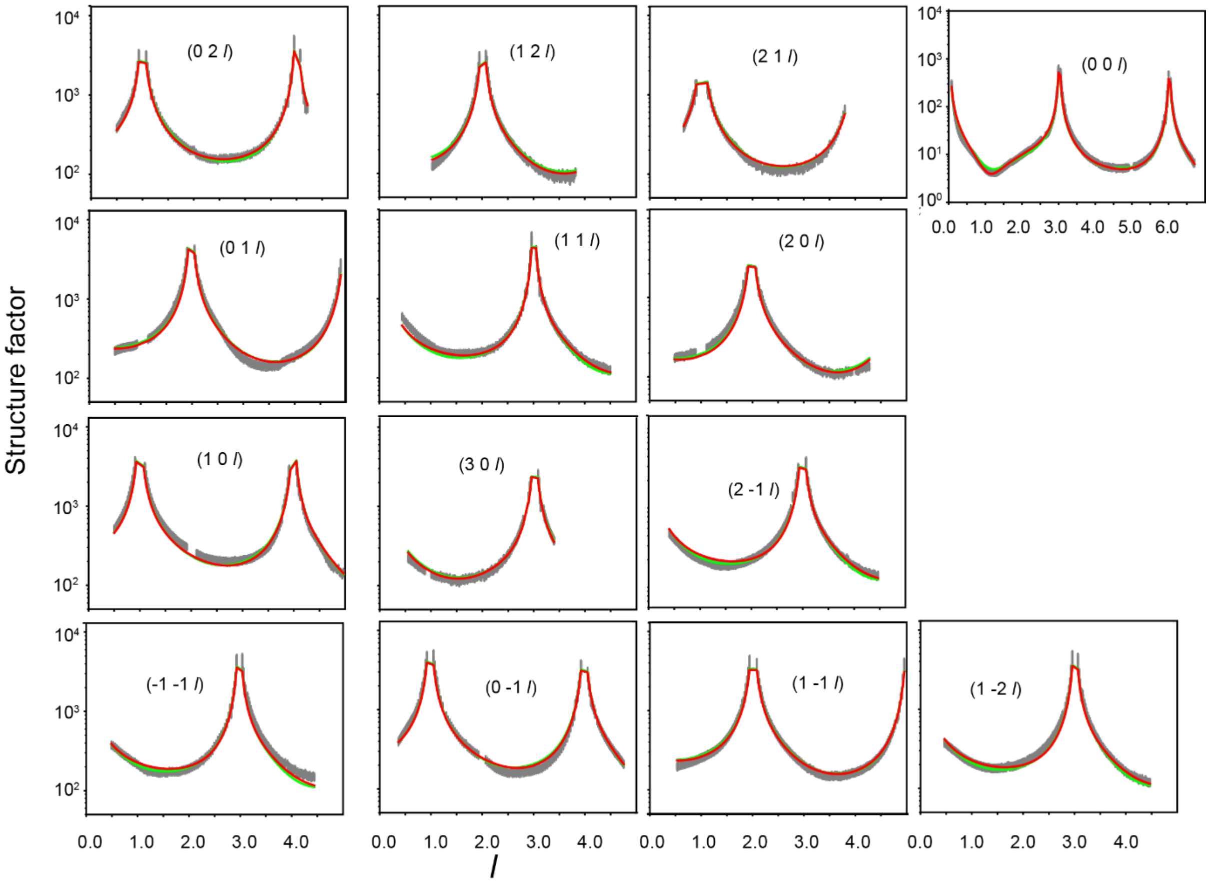

Figure S2 Experimental CTRs (grey data points) compared with the results of simulations for the best-fit adatom model (red curves) and the best-fit no-adatom model (green curves).

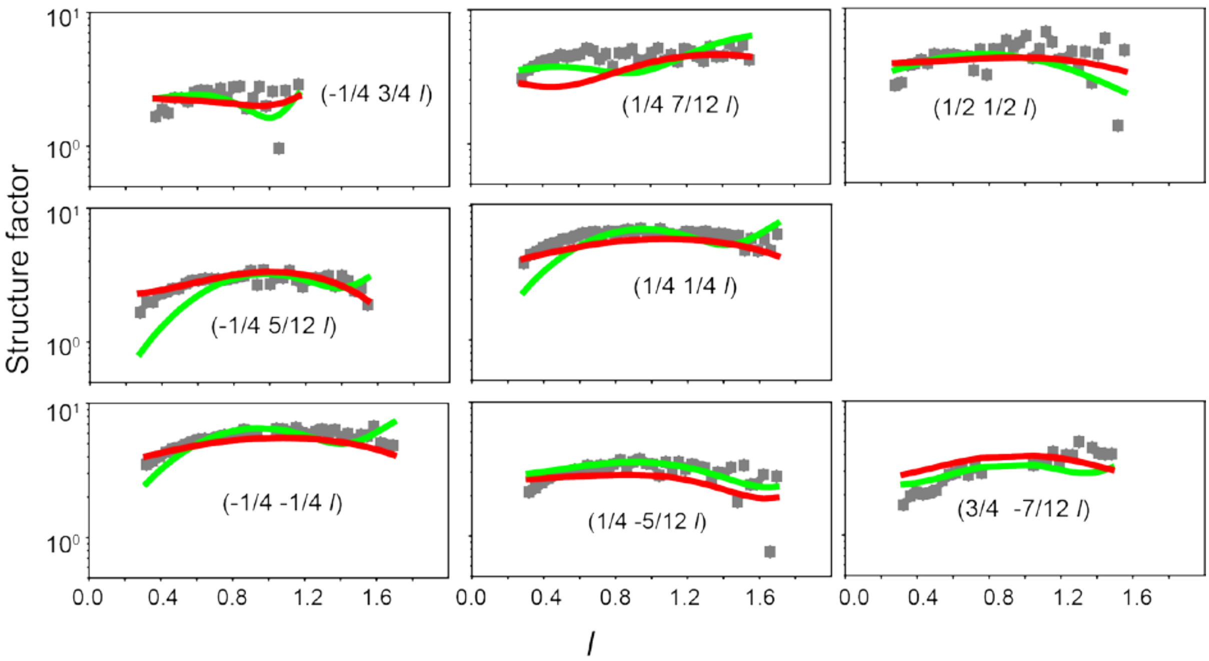

Figure S3 Experimental FORs (grey data points) compared with the results of simulations for the best-fit adatom model (red curves) and the best-fit no-adatom model (green curves).

Two-adatom structural model

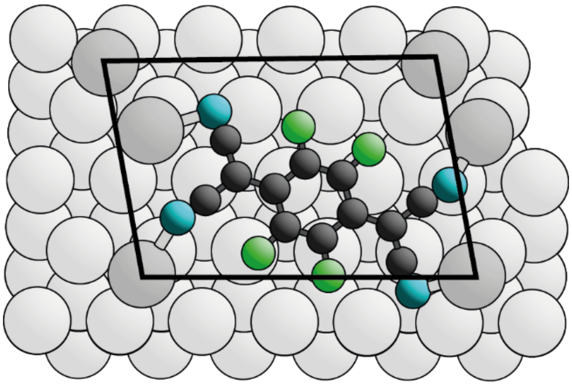

Figure S4 Plan view of the two-adatom model of the Ag(111) $\begin{bmatrix} 4 & 0 \\ 1 & 3 \end{bmatrix}$ F_4TCNQ structure giving the lowest value of χ^2 by SXRD. Atom colouring as in Figure 6 of the main manuscript.
